# Food insecurity and risk of cholera: A cross-sectional study and exploratory analysis of potential mediators

**DOI:** 10.1371/journal.pntd.0010574

**Published:** 2023-02-06

**Authors:** Ahmed D. Elnaiem, Molly F. Franke, Aaron Richterman, Yodeline Guillaume, Kenia Vissieres, Gertrude Cene Augustin, Ralph Ternier, Louise C. Ivers

**Affiliations:** 1 Department of Medicine, Division of Global Health Equity, Brigham and Women’s Hospital, Boston, Massachusetts, United States of America; 2 Department of Global Health and Social Medicine, Harvard Medical School, Boston, Massachusetts, United States of America; 3 Division of Infectious Diseases, Hospital of the University of Pennsylvania, Philadelphia, Pennsylvania, United States of America; 4 Center for Global Health, Massachusetts General Hospital, Boston, Massachusetts, United States of America; 5 Zanmi Lasante, Partners In Health, Cange, Haiti; Tufts Medical Center, UNITED STATES

## Abstract

**Background:**

Food insecurity has been independently associated with developing cholera and there is an inverse relationship between national food security and annual cholera incidence. However, the factors that mediate the risk of cholera among food insecure households remain largely unexplored.

**Methodology and principal findings:**

In a cross-sectional survey of rural households in Haiti, we explored the role of food behaviors (i.e., dietary choices and food-handling practices) as mediators of cholera risk among food-insecure families. We generated a series of multivariable regression models to test hypothesized associations between the severity of food insecurity (measured by the Household Hunger Scale), hygiene and food behaviors, and history of severe, medically-attended cholera.

Moderate household hunger (Adjusted Odds Ratio [AOR] 1.47, 95% Confidence Interval (CI) 1.05–2.04; p = 0.021) and severe hunger (AOR 2.45, 95% CI 1.45–4.15; p = 0.001) were positively associated with a history of severe, medically-attended cholera compared with little to no household hunger. Household hunger was positively associated with three behaviors: antacid use, consumption of leftover non-reheated food, and eating food and beverages prepared outside of the home (i.e., at a restaurant or from a vendor). Consumption of outside food items and antacid use were positively associated with a history of cholera.

**Conclusion:**

Our findings suggest that food behaviors may mediate the association between food insecurity and cholera and contribute to an understanding of how interventions could be designed to target food insecurity as part of cholera prevention and control.

## Author summary

Food insecurity has been found to be a risk factor for cholera at the household and national level, however the mechanism through which food insecurity may increase the risk of cholera remains unknown.

In a large cross-sectional survey of 1072 households in rural Haiti, we observed a robust independent association between food insecurity—defined as a persistent lack of access to food in adequate quantity or quality and measured by the Household Hunger Scale—and reported history of severe, medically-attended cholera. The association was dose-dependent, with the odds of reported cholera increasing linearly with household food insecurity. Household food insecurity was associated with three high-risk behaviors: antacid use, consumption of leftover non-reheated food, and eating food and beverages prepared outside of the home. Two high-risk behaviors—including antacid use and consumption of food and beverages prepared outside of the home (i.e., at a restaurant or from a vendor)—were independent risk factors for cholera.

High-risk food practices may be one causal pathway by which food insecurity increases risk of developing cholera. In this study, we provide a framework for future longitudinal and qualitative studies to investigate whether interventions that target food insecurity could also reduce cholera risk among populations who face a high burden of both conditions.

## Introduction

Cholera and food insecurity are afflictions of inequity that sicken and kill impoverished and vulnerable populations. In 2018, an estimated 1.3 to 4.0 million cases and 21,000 to 143,000 deaths from cholera were reported worldwide [1] and globally cholera outbreaks have been increasing [2]. Meanwhile, in 2020, an estimated 2 billion people faced moderate or severe food insecurity [3]. In poor communities worldwide, close links between determinants of access to food, clean water, and basic sanitation give rise to significant overlaps in the distribution of food insecurity and cholera, particularity in humanitarian emergencies and conflict zones.

Food insecurity is a multidimensional phenomenon defined by an *uncertain* or *limited availability* of nutritionally adequate or safe food [4]. Over the past two decades, a growing body of literature has identified direct relationships between food insecurity and adverse health outcomes including in patients with metabolic disease [5–9], infectious diseases such as HIV and tuberculosis [10], cardiovascular disease [11,12], sleep disorders [13], and mental health conditions such as depression, anxiety, and psychological distress [14].

Because of the well-documented relationship between food insecurity and poor health outcomes in other diseases, and the frequent co-occurrence of cholera and food insecurity in impoverished settings, we have recently begun to evaluate the potential role of food security in the risk of developing cholera. We previously found that food insecurity was associated with a risk of developing cholera and a risk of death from cholera [15,16]. In a subsequent retrospective analysis of data from 30 countries, we demonstrated an inverse relationship between national food security, as measured by the Global Food Security Index, and the annual incidence of cholera [17].

Food insecurity may affect an individual’s risk of acquiring *Vibrio cholerae* or of developing severe cholera when infected with the pathogen through several hypothesized pathways. Firstly, malnutrition may impair the immune system, reducing gut barrier function and allowing *V*. *cholerae* to thrive, resulting in more severe symptomatic illness than might have otherwise occurred [18–20]. Secondly, decreased food availability may negatively impact food-related behavior, or lead to suboptimal food hygiene practices as an individual’s risk-benefit estimation on what they eat, where they obtain food, or how they prepare food may be impacted when their attention is directed toward their most immediate food needs [4,21]. Gastrointestinal symptoms of hunger may result in use of medications or other remedies to alleviate discomfort. Lastly, poor mental health arising from food insecurity may undermine individual and community resilience to the economic losses and emotional strains caused by outbreaks of cholera [21]. Evidence evaluating the pathways by which these factors may contribute to an increased risk of symptomatic cholera among those who are food insecure is limited [22–25].

To address this knowledge gap, we assessed the potential role of household food behaviors, i.e., dietary choices and food-handling practices, as potential mediators of the relationship between food insecurity and cholera using a cross-sectional survey of rural Haitian households. In this study, we hypothesized that food insecurity would be positively associated with household behaviors—specifically antacid use and consumption of leftover non-reheated food, raw fruits or vegetables, raw or undercooked fish, or food and beverages prepared outside of the home—that would predispose individuals to develop severe, medically-attended cholera, and that these behaviors would be positively associated with severe cholera.

## Methods

### Ethics statement

Institutional Review Board approval was received from Partners Healthcare (Protocol #2018P001220) & Zanmi Lasante, Haiti (Protocol #128). All participants provided verbal informed consent to participate in the study.

### Selection and description of participants

We administered a cross-sectional survey to a random sample of 1072 households in the town of Mirebalais in Central Haiti between July and August, 2019. Households were randomly selected from data collected in a 2017 census that had enumerated 27,911 household and was conducted by our team as part of a public health program aimed at prevention and control of cholera [26]. A total of 1129 households were invited to participate in the study. Of these, we excluded a total of 57 households (9 declined to participate, 20 could not be contacted, and 28 did not reside permanently in the study’s catchment area of Mirebalais).

A household was defined as an individual or group of related or unrelated individuals that slept at least half the week under the same roof, and shared resources such as food or cost of living expenses. Respondents were primarily adult heads of the household who were eighteen years or older and provided written informed consent. If the head of a household was unavailable, we surveyed another adult member of the household who could answer the survey questions about the household. After the interview, and with consent of the respondent, the study worker observed water, sanitation, and hygiene facilities at the household. Survey instruments were translated, and back-translated by bilingual research team members in English and Haitian Creole and had previously been extensively field-tested in the study population in central Haiti [16,27].

The survey was comprised of questions on household demographics, livelihood, assets, knowledge of and practices relating to cholera transmission, diet, medication use, and food-handling practices over the week and month prior to the interview. Household food insecurity was measured using the Household Hunger Scale (HHS), a cross-culturally validated indicator consisting of three items drawn from the Household Food Insecurity Access Scale [28]. Households were classified into three categories based on established thresholds: little to no hunger, moderate hunger, and severe hunger in the household. A history of severe, medically attended cholera was obtained by self-report through two questions a) "Have you had diarrhea requiring you to stay overnight in a cholera treatment center or hospital since 2010?", and b) "Has anyone in your household (besides you) ever spent the night in a cholera treatment unit?". We asked respondents if they had been vaccinated against cholera, and if yes, requested to confirm this by reviewing a vaccination card. We estimated household poverty status using the Simple Poverty Scorecard—a validated poverty assessment tool specific to Haiti based on 11 indicators [29]. The scorecard generates a numeric poverty score (continuous variable) and estimates the likelihood of consumption below a poverty line (about $1/day)—a lower score indicates higher relative poverty.

### Statistical analysis and causal framework

**Fig 1:**
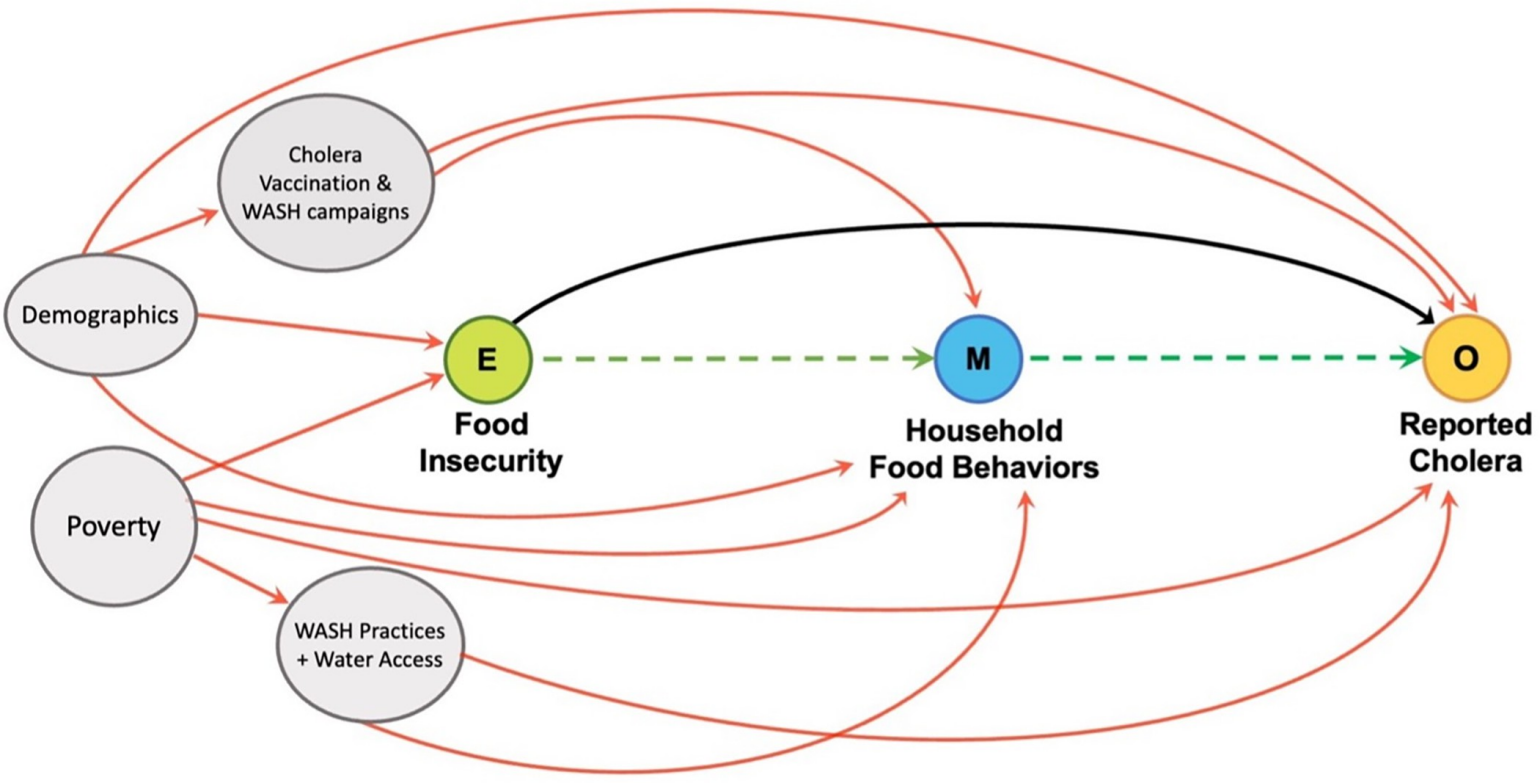
Directed acyclic graph illustrating hypothesized relationship pathways. Exposure is shown in green, mediating food behaviors in light blue, confounders in grey, and outcome in yellow. The indirect effect of food insecurity occurring through mediating household food behaviors is shown in green (dashed line). The direct effect of food insecurity on cholera is shown in black, and that of confounders in red.

We first constructed a directed acyclic graph, based on published risk factors for cholera and biological plausibility, to guide our exploratory analysis and illustrate our hypothesized causal framework (Fig 1). We used logistic regression models to assess the relationship between household food insecurity and history of cholera. We generated an initial univariate model to calculate the crude unadjusted odds ratio (OR) with a 95% confidence interval (CI) between household hunger—the Household Hunger Scale was modeled as moderate and severe food insecurity versus little to none [28]—and history of severe, medically-attended cholera in the household. We then performed multivariable analyses to calculate an adjusted odds ratio (AOR) and 95% CI. In a secondary analysis, we modeled linear categories of household hunger (none, mild, moderate, severe) against medically-attended cholera and performed a log-likelihood ratio test to assess whether this linear categorization fit the data best.

We selected confounders based on our causal framework, prior publications of risk factors for cholera, and observed associations in our data. These included: poverty score, single marital status (head of household), cholera vaccination status, improved household water source, money spent on water (0, 0–15, 15–30, and >30 Haitian gourdes), distance to a water source (<15 min, 15–30 min, 30-45min, 45 min– 1 hour, and >1 hour) and latrine use [16,27,30,31]. We treated prior cholera vaccination and variables related to knowledge of handwashing as confounders of cholera in respondents. We based this decision on evidence from a previous longitudinal study conducted in Haiti that demonstrated that a cholera vaccination campaign was associated with increased handwashing knowledge and practice [31].

To interrogate the mediating effects of our hypothesized food behavior pathway, we utilized a two-step approach using logistic regression models. These models identified whether food insecurity was associated with specific high-risk behaviors in a household, and whether these behaviors were associated with cholera. First, we used multivariate regression models to estimate the association between food insecurity and specific behaviors. We considered five variables: consumption of leftover unheated food, raw fruits/vegetables, raw or undercooked fish, foods prepared outside the home, and antacid use over the prior week. We ran separate multivariable regression models, with each of the five food behavior variables as the dependent variable, household food insecurity as the independent variable, and the following confounders based on our causal framework and observed associations in the data: poverty score, single marital status, prior cholera vaccination, and knowledge of handwashing.

We then used multivariate regression models to assess the association between specific food behavior variables and history of household cholera. We adjusted for the same cholera risk factors included in the overall model estimating the association between food insecurity and cholera. To evaluate for multicollinearity in multivariate models, we calculated a variance inflation factor among covariates for each model—a factor greater than 2.50 was deemed indicative of multicollinearity.

There were missing data for only two variables, presence of a household latrine and covered water storage vessel, both of which were collected via observation of household WASH infrastructure. We used the missing indicator method to account for missingness in these two variables.

All quantitative analyses were performed using R Studio version 1.2.1335 for Mac with R version 3.6.1 for Mac.

## Results

A total of 1072 households, with a median size of 5 individuals (Interquartile Range 3–6), consented to participate in the survey (Table 1). Among all respondents, 52% were female, 60% worked primarily in agriculture, 42% never attended school, and 27% were single-parent heads of household. Approximately one-third of households (n = 343) reported at least one instance of severe, medically-attended cholera. Most households (64.4%) experienced either moderate or severe levels of household hunger.

**Table 1:** Household characteristics, dietary, food, and hygiene practices and knowledge of cholera in a cross-sectional household survey of Mirebalais, Haiti 2019 (N = 1072).

			Little to no Hunger ** N = 382		Moderate Hunger ** N = 598		Severe Hunger ** N = 92	
			n	%	n	%	n	%
**Household Characteristics**								
Household size [median (25^th^ -75^th^ percentile)]			4 (3–6)		5 (3–6)		5 (4–7)	
Number of children < 5 years old			108	28%	213	36%	42	46%
Single marital status (head of household)			120	31%	203	34%	34	37%
Poverty score [median (standard deviation)]			48 (15.9)		40 (15.1)		31 (14)	
Likelihood of consumption below national poverty line [27]			44.8%		62.7%		83.6%	
Household history of severe, medically-attended cholera			85	22%	211	35%	47	51%
**Dietary and Other Intake Practices** ^*^								
Antacid use			174	46%	258	43%	61	66%
Family was unable to reheat leftover food before eating			78	20%	121	20%	43	47%
Consumed food/beverage prepared outside of the home			121	32%	280	47%	60	65%
Source of procured item(s)		Family/friend’s house	48	40%	162	58%	46	77%
		Restaurant		29%	68	24%	6	10%
		Marketplace	37	31%	99	35%	32	53%
Dietary composition and medication use		Raw fruits and vegetables	210	55%	271	45%	40	43%
		Raw or undercooked fish	11	3%	17	3%	5	5%
		Almost daily fish or seafood consumption	27	38%	12	55%	3	73%
		Leftover unheated rice	71	19%	124	21%	48	52%
		Beverage with ice obtained outside of household	288	75%	407	68%	76	83%
**Water, Sanitation and Hygiene Knowledge and Reported Practices**								
Utilization of an improved household water source^**†**^			190	50%	230	38%	30	33%
Water from usual source was unavailable at least 1 whole day in the past month			153	40%	326	55%	44	48%
Always treats household water			272	71%	247	41%	30	33%
	Reason for not always treating water	Too expensive	4	1%	54	15%	0	0%
		We don’t have what we need to treat	54	49%	208	59%	46	74%
		Hard to get what we need to treat	27	25%	114	32%	35	56%
Time to fetch water and return by foot		<15 min	204	53%	234	39%	40	43%
		15–29 min	69	18%	128	21%	24	26%
		30–59 min	58	15%	160	27%	16	17%
		1 hour +	51	13%	76	13%	12	13%
Money spent daily on water^**#**^		None	192	50%	440	74%	73	79%
		0–30 gourdes	119	31%	84	14%	9	10%
		> 30 gourdes	71	19%	74	12%	10	11%
Washes hands with soap and water > = 4 times daily			261	68%	280	47%	68	74%
Soap always available in the home			241	63%	206	34%	29	32%
Offers this example of when hand washing is appropriate		After the toilet	262	69%	476	80%	77	84%
		Before eating	332	87%	517	86%	78	85%
		Before preparing food	166	43%	170	28%	22	24%
		After touching things like money, telephones, etc.	147	38%	119	20%	30	33%
		After handling a person with vomiting or diarrhea	71	19%	82	14%	9	10%
Washes hands with soap and water > = 4 times daily			261	68%	280	47%	68	74%
Soap always available in the home			241	63%	206	34%	29	32%
Use of covered water storage vessel ^††^			204	85%	380	81%	61	79%
Household toilet system ^†††^		Flushing toilet	48	17%	13	3%	3	4%
		Improved pit latrine	106	38%	120	25%	9	12%
		Unimproved pit latrine	112	40%	268	56%	53	73%
		Open area or garden	10	4%	60	13%	7	10%
		Composting toilet or bucket	4	1%	14	3%	1	1%
Opening of water storage vessel ^††^		Narrow-mouthed (hand cannot fit in)	125	52%	163	35%	30	39%
		Wide-mouthed (hand can fit in)	116	48%	309	66%	47	61%

* Primary survey respondents (head of household) were asked about consumption over the prior week.

** Household Hunger Scale scored as: little to no hunger in the household (score 0–1), moderate hunger in the household (score 2–3), or severe hunger in the household (score 4–6).

† Improved water sources were defined by Joint Monitoring Programme for Water Supply and Sanitation (JMP) standards and included: piped household water, protected wells or springs, bottled water, and collected rainwater [32].

# At the time of the study: $1 USD = G 94.1376; 30 gourdes was approximately $0.32 USD.

†† Missing water storage vessel observations exist for 282 households.

††† Missing household toilet system observations for 244 household.

The distribution of socioeconomic characteristics, food behaviors, and WASH knowledge and reported practices varied significantly by the severity of household hunger (Table 1). In the week before the interview, moderately and severely hungry households were more likely to have consumed food or beverages prepared outside and been unable to reheat their food. Households with severe hunger reported more frequent use of antacids (66% vs. 43% in moderate, and 46% in little to no hunger; p<0.0001) and were least likely to report always treating their water (33%, p<0.0001). Households with moderate and severe hunger were less likely to spend money on water (p<0.0001), collect from an improved water source (p = 0.0003), or travel less than 15 minutes to fetch water and return (p<0.0001). These households were also more likely to store water in a wide-mouthed container in which a hand could fit (66% for moderate; 61% for severe) compared to those with little to no hunger (48%).

### Association of food insecurity and cholera

Univariate analysis demonstrated a significant association between moderate (OR 1.91, 95% CI 1.42–2 .56; p<0.001) and severe (OR 3.65, 95% CI 2.27–5.88; p<0.001) household hunger and history of cholera relative to little to no household hunger (Table 2). Multivariable analysis including hypothesized confounders yielded a similar, although attenuated, association for moderate (AOR 1.47, 95% CI 1.05–2.04; p = 0.021) and severe (AOR 2.45, 95% CI 1.45–4.15; p = 0.001) hunger in the household.

The relationship between household hunger and reported cholera was linear (likelihood ratio p-value = 0.9997): we observed a 40% increase in the odds of cholera with each increase in household food insecurity category (AOR 1.53, 95% CI 1.06–1.84; p = 0.0006).

**Table 2:** Unadjusted univariate and adjusted multivariable associations between household food insecurity and history of severe, medically-attended cholera in a cross-sectional household survey of Mirebalais, Haiti 2019 (N = 1072).

	Unadjusted Univariate Model			Adjusted Multivariable Model ^a^		
	Odds ratio	95% CI	p-value	Odds ratio	95% CI	p-value
Little to no hunger	ref			ref		
Moderate hunger	1.91	1.42–2.56	<0.001	1.47	1.05–2.04	0.022
Severe hunger	3.65	2.27–5.88	<0.001	2.45	1.45–4.15	0.006

(a) covariates include poverty score, single marital status, vaccination, improved water source, household latrine, covered water storage vessel, money spent daily on water, distance to a water source, and handwashing knowledge.

### Household food insecurity as a predictor of high-risk behaviors

Household food insecurity was positively associated with three high-risk behaviors: consumption of food or beverages prepared outside of the home (moderate: AOR 1.92, 95 CI 1.45–2.5, p<0.0001; severe: AOR 4.32, 95 CI 2.61–7.28, p<0.0001), antacid use (severe: AOR 2.51, 95 CI 1.52–4.19; p = 0.0004), and consumption of leftover non-reheated food (severe: AOR 4.35, 95 CI 2.57–7.38, p<0.0001) (Table 3).

**Table 3:** Adjusted associations between household food insecurity and behaviors in a cross-sectional study of 1072 households in Mirebalais, Haiti, 2019.

	Little to no Hunger			Moderate Hunger			Severe Hunger		
**High-Risk Behavior** ^a^^*^	AOR	95% CI	p-value	AOR	95% CI	p-value	AOR	95% CI	p-value
Antacid use	ref			0.95	0.72–1.25	0.7041	2.51	1.52–4.19	0.0004
Raw fruits/vegetables	ref			0.69	0.52–0.92	0.0107	0.72	0.43–1.17	0.1868
Undercooked fish	ref			1.13	0.51–2.64	0.7610	2.48	0.71–7.76	0.1290
Unable to reheat food	ref			1.10	0.79–1.56	0.5569	4.35	2.57–7.38	<0.0001
Consumes outside foods	ref			1.92	1.45–2.57	<0.0001	4.32	2.61–7.28	<0.0001

(a) Covariates: poverty score, single marital status, cholera vaccination, and knowledge of handwashing.

* Consumed over the prior week

### Association of household behaviors and cholera

**Fig 2:**
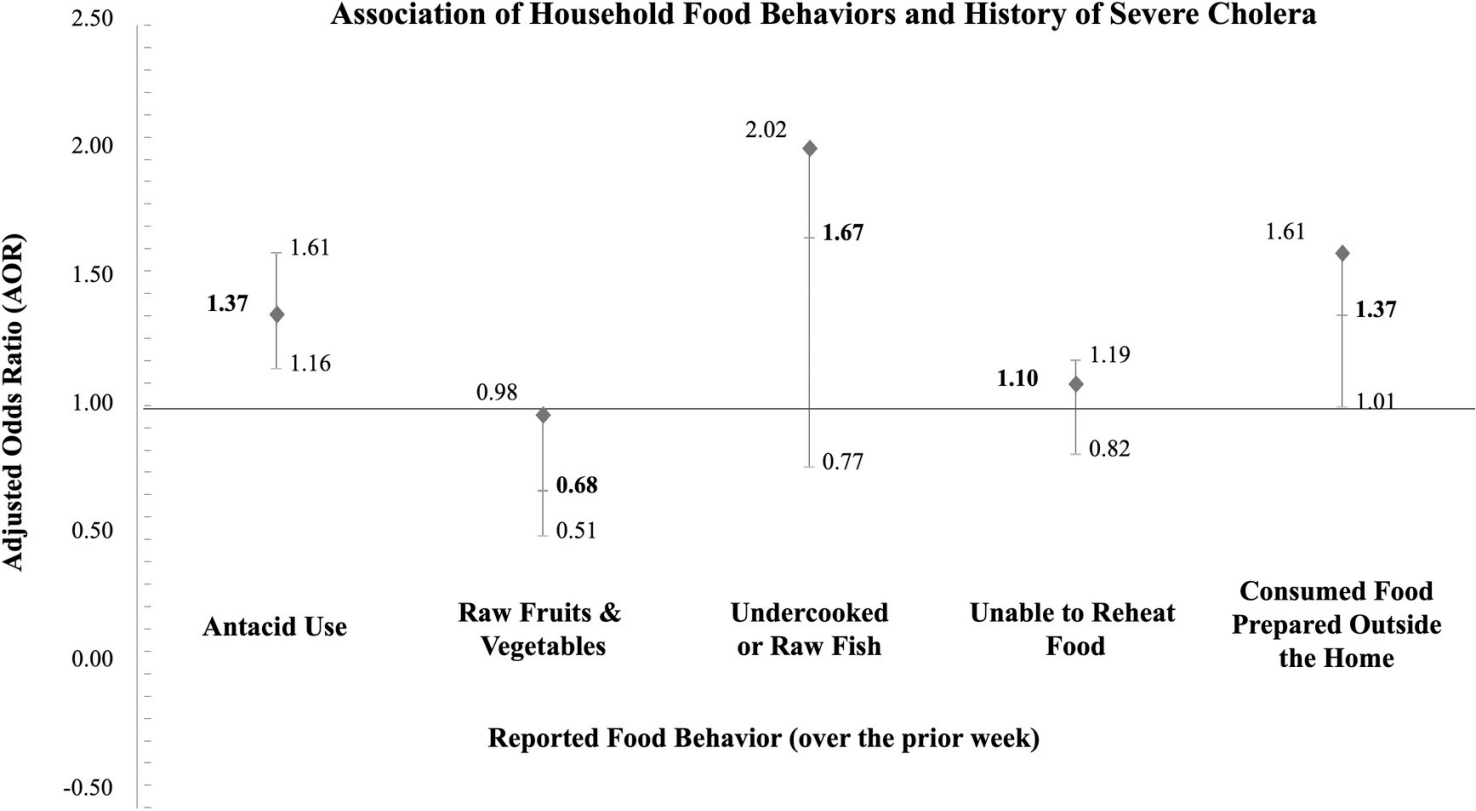
Adjusted associations of high-risk behaviors and history of severe medically-attended cholera in a cross-sectional study in Mirebalais, Haiti 2019 (N = 1072).

Fig 2 reports the associations between high-risk household behaviors and history of severe, medically-attended cholera. In adjusted analyses, we found an independent association between household history of cholera and consumption of outside food (AOR 1.61; 95% CI 1.16–2.86 p = 0.046) and antacid use (AOR 1.34; 95% CI 1.06–1.16, p = 0.00472) over the prior week. Consumption of raw fruits and vegetables was protective of household history of severe medically-attended cholera (AOR 0.68, 95% CI 0.51–0.98, p = 0. 0483).

## Discussion

In this cross-sectional study of 1072 households in central Haiti, we found a positive association between food insecurity and self-reported severe medically-attended cholera. We provide evidence that specific, high-risk behaviors serve as potential mediators of this association. Our findings illuminate one possible causal pathway through which food insecurity may predispose individuals to infection with the causative organism of cholera; in doing so, our findings have important implications for cholera prediction, response and control strategies [15,17].

We found that households with moderate and severe food insecurity were more likely to report high-risk behaviors for cholera. These included more frequent antacid use, consumption of food prepared outside the home, and eating of unheated leftover food items. Two of these behaviors—antacid use and consumption of food prepared outside the home—were also risk factors for cholera. A third risk factor, consumption of undercooked fish, was marginally associated with both food insecurity and cholera. These findings are consistent with a prior systematic review and metanalysis which identified all three behaviors as risk factors for cholera [30]. These behaviors likely increase the risk of infection with cholera through more frequent household contact with contaminated foods and beverages.

In both high- and low-income contexts, hunger has been shown to lead to high-risk food handling practices. One study found that individuals experiencing food insecurity in the U.S. (New Jersey) needed to make pressured choices while scavenging for meals, which resulted in the consumption of contaminated food [33]. For example, participants reported consuming food left over by others as well as food items that required removal of spoiled areas with slime, mold, or insects [33]. Coping strategies are also context-specific and influenced by environmental, socioeconomic, and cultural factors [34]. However, consistent themes emerge across studies as insecure access to food has been tied to alterations in the quantity and types of foods consumed, nonfood spending allocations (e.g., reductions in medical spending), and overall behavioral patterns (e.g., forms of employment which may increase risks for sexually transmitted infections) [34–38]. Qualitative research into informal coping strategies has identified specific examples to include: stretching and substitution techniques, such as using water in place of milk in breakfast cereals; intake of expired and nearly expired foods; reductions in meal size, diversity, and frequency; transitioning to less expensive foods; and making one large pot of food to consume for several days [34–38]. In a study from Mali, the inability to afford cooking fuel for reheating and cooking was identified as risk factor for cholera as it increased the likelihood of eating cold, contaminated foods [23].

The use of antacids is a well-established risk factor for developing cholera after exposure to *V*. *cholerae* [39]. It has been reported in several contexts that individuals who are chronically hungry may also experience chronic gastrointestinal complaints as a result of food insecurity [40–42]. Medications that suppress gastric acidity are readily available at low cost and offer utility in treating a range of symptoms, leaving antacids as a plausible choice in response to gastrointestinal complaints among those who are food insecure. High rates of dyspepsia and infection with *Helicobacter pylori* have been reported in Haiti [43], as well as evidence of antibiotics, H2 receptor antagonists, and antacids being readily dispensed for their treatment [44,45].

A limitation of our exploratory study is its cross-sectional design that precludes discernment of temporality. For instance, it is plausible that a household’s experience of cholera may subsequently increase their risk of food insecurity through costs associated with hospitalization, loss of income, and funerals [46,47]. While we cannot exclude the possibility that food insecurity occurred as a consequence of cholera infection, it is unlikely since high levels of food insecurity existed in Haiti well before the cholera epidemic began and has historically been driven by high food and fuel prices, droughts, and socio-political instability [3]. Moreover, it is unlikely that cholera would directly lead to the high-risk household food behaviors we evaluated, independently of household hunger. Regardless, the potential mediating role of food behaviors in increasing cholera risk among individuals with household hunger should be confirmed in longitudinal research that incorporates repeated measurements of households over time. A second limitation of our study is that cholera was self-reported because reliable medical records were not available to confirm cholera cases. Our study was limited to one region in Haiti, and future studies could investigate the generalizability of our findings by extending this work, including to other geographical regions where cholera is endemic or epidemic and where food insecurity is also high.

## Conclusion

As efforts are underway to eliminate cholera transmission and reduce cholera deaths by 2030, innovative public health approaches are needed to complement the backbone of water, sanitation, hygiene and vaccination programs. This analysis furthers our understanding of the relationship between food insecurity and the risk of developing cholera and provides a framework for putative longitudinal and qualitative studies to investigate whether interventions that target food insecurity could also reduce cholera risk among populations that face a high burden of both conditions. Cholera impacts the worlds most impoverished communities, and inequity in access to basic human necessities such as water, sanitation, hygiene, and food are important components of the complex, intersectoral risk for ill-health.
